# Social-Aware Driver Assistance Systems for City Traffic in Shared Spaces

**DOI:** 10.3390/s19020221

**Published:** 2019-01-09

**Authors:** Alberto Fernández-Isabel, Rubén Fuentes-Fernández

**Affiliations:** 1 Data Science Laboratory (DSLab), Universidad Rey Juan Carlos, 28933 Móstoles, Spain; alberto.fernandez.isabel@urjc.es; 2 Research Group on Agent-Based, Social & Interdisciplinary Applications (GRASIA), Universidad Complutense de Madrid, 28040 Madrid, Spain

**Keywords:** shared space, multi-modal traffic, people displacement, traffic social property, social knowledge, Social-Aware Driver Assistance System, Multi-Agent System

## Abstract

Shared spaces are gaining presence in cities, where a variety of players and mobility types (pedestrians, bicycles, motorcycles, and cars) move without specifically delimited areas. This makes the traffic they comprise challenging for automated systems. The information traditionally considered (e.g., streets, and obstacle positions and speeds) is not enough to build suitable models of the environment. The required explanatory and anticipation capabilities need additional information to improve them. *Social aspects* (e.g., goal of the displacement, companion, or available time) should be considered, as they have a strong influence on how people move and interact with the environment. This paper presents the Social-Aware Driver Assistance System (SADAS) approach to integrate this information into traffic systems. It relies on a domain-specific modelling language for social contexts and their changes. Specifications compliant with it describe social and system information, their links, and how to process them. *Traffic social properties* are the formalization within the language of relevant knowledge extracted from literature to interpret information. A multi-agent system architecture manages these specifications and additional processing resources. A SADAS can be connected to other parts of traffic systems by means of subscription-notification mechanisms. The case study to illustrate the approach applies social knowledge to predict people’s movements. It considers a distributed system for obstacle detection and tracking, and the intelligent management of traffic signals.

## 1. Introduction

City traffic is changing worldwide. Policies are designed to reduce the number of conventional fuel vehicles in city centers, to reduce pollution levels and give more space to other traffic participants [1]. This should make it possible to improve public transport services so that people use them more. In addition, it is creating opportunities for the use of less contaminating means of transport (e.g., bicycles, scooters, and electric cars). These can better satisfy users’ requirements regarding the environment, health, and costs, while circumventing policies to restrict access to some city areas. Frequently, they also offer mobility which is more tailored to individual needs, with vehicles smaller than cars or motorbikes. These vehicles are appearing in large numbers in cities, often as part of vehicle-sharing services [2,3].

With these trends, we are seeing a rise in the number of areas with shared spaces [4]. These are characterized by the blurring of barriers to segregate different types of traffic, for instance, areas with shared paths or painted lanes. This produces multi-modal traffic flows where interactions among participants become more complex [5], as there is a greater variety of perceptions to evaluate regarding traffic and the environment, the capabilities of vehicles, people’ characteristics, and situations.

These shared environments make it necessary to reconsider the design of Driver Assistance Systems (DASs) [6] and Intelligent Transportation Systems (ITSs) [7]. These aim to enhance different aspects of traffic and travelling, such as active and integrated safety, fuel and energy consumption, and vehicle flows. For this purpose, they use different types of information, e.g., type and location of traffic signals, lanes, and the position and speed of obstacles. However, this “traditional” information does not seem to be enough in shared spaces, where methods of movement are heterogeneous, distances between participants are short, and trajectories can suddenly change. Here, the systems need more detailed information to provide their services.

One of the potential new types of information are the *social aspects* considered in this work. These include factors that affect people’s behaviors and depend on their personal characteristics (e.g., gender, age, and capabilities), the surrounding people (e.g., type of companion or crowd), the resources they use (e.g., type of vehicle and mobile phones), and the context (e.g., activity and meaning of the environment). The literature already acknowledges the influence of these aspects on traffic (e.g., pedestrians in groups [8], bicycles [9], or passengers [10]).However, there are few studies that focus on how to incorporate them into traffic systems, and most of them adopt ad-hoc approaches [6,7]. This makes it difficult to integrate systems or different information types to provide more sophisticated services.

The current study develops a framework for Social-Aware DASs (SADASs). These are intended to facilitate the inclusion of the previous social aspects in traffic systems. This is achieved through mechanisms for flexible management of that information, which can be adapted to different requirements and integrated with other information and components. This social information allows traffic systems to better understand their context and how different actors and elements can behave accordingly, thus improving their decision-making.

The SADAS framework has two components. The first one is a Modelling Language (ML) to specify social information and its transformation. The second one is the architecture that implements that processing and determines the interfaces to interact with the other elements in traffic systems.

The ML for SADAS (i.e., the SADAS-ML) is based on two previous studies. First, a general framework with an ML (i.e., the ITSML) to model and simulate ITSs [11]. Its concepts include among others *sensor* and *actuator*, *agent* and *person*, *vehicle*, and *information* and *method*. Second, a framework to model social systems in engineering contexts based on the concept of social activity [12].

The SADAS-ML extends the ITSML with new concepts to model *social information* and its changes. For instance, the types for low-level sensor *data* and *social information*, *physical objects* and their characteristics, and *processes* such as social *activities* and information *transformations*. The language also introduces reliability attributes to indicate different degrees of certainty about information.

In this context, reusable knowledge is characterized as *traffic social properties*. This follows the studies on *social properties* [12] as patterns on social information for software engineering. In SADASs, the properties describe prototypical information applicable in a wide range of traffic situations. For instance, that near a hospital there could be people with reduced mobility. This knowledge is usually extracted from literature, reports, and statistics. The properties are specified following the template for the original *social properties* [12], though using concepts and diagrams that conform to the SADAS-ML. Here, the discussion uses only the diagrams in these templates.

The framework also defines an architecture to work with the social information. It corresponds to a Multi-Agent System (MAS) [13] with several specific *social components*. These include: *sensing agents* to transform low-level data from sensors into information; *reasoner agents* to derive new information from that available; *observer agents* that send the information to the rest of the traffic system and support external queries. These agents use specific resources to support their functionality, such as a social engine to derive information using processes. This kind of architecture follows common practices in information fusion [14].

The processing of this social information uses a pattern-matching algorithm. It looks for instances of the query specifications among available information. When it is successful, it applies the processes linked to the query. For example, this allows it to be checked that current information meets some property, or to derive new information.

The case study that illustrates the application of the framework integrates two studies and extends them with a SADAS to improve their services. The first study is an ITS [15] for the adaptive control of traffic signals using a hierarchical control strategy. It includes sensors such as cameras and inductive loops. The second one is a DAS [16] that supports the distributed detection of pedestrians from cars with cameras. The use of cameras in both systems allows characteristics to be extracted from the environment and participants in traffic [17,18]. The SADAS uses this information to derive new information on people’s potential behaviors given their activities. This allows traffic signals and vehicle warnings to be adjusted for safer and more fluid traffic than in the original studies. This example is complemented with an additional discussion on other case studies.

The rest of the paper is organized as follows. Section 2 introduces the background on the ITS framework [11] and social properties [12]. Section 3 describes the components of the SADAS framework. The experimentation in Section 4 illustrates the use of the approach with the case study and contains an extended discussion. Section 5 compares the framework with related work. Finally, Section 6 discusses some conclusions and future work.

## 2. Background

The SADAS framework appears in the context of traffic systems and the management of social information. Its components and the information models relating to traffic are described using extensions of the concepts in the general modelling framework for ITSs in [11] (see Section 2.1). The description of social aspects extends the work on social properties [12] (see Section 2.2).

### 2.1. ITS Modelling and Simulation Framework

This framework aims to facilitate the Model-Driven Engineering (MDE) of simulations of ITSs [11]. In MDE approaches [19], development artefacts (e.g., code, documentation, and tests) are mostly generated from system specifications (i.e., models) using semi-automated transformations. In the case of this framework, such specifications are compliant with the ITSML. Its conceptual framework is based on the Driver-Vehicle-Environment (DVE) approach [20].

The base concept of the ITSML is the *component*, which represents a general element of an ITS or the environment. It has an internal state defined as *attributes*, and interfaces characterized as *methods* (e.g., consult, modify and act). Control and information flows among components use those methods. A component can also have other components as parts.

Attributes, and the parameters and results of methods, are defined as pieces of *information*. They have a name, a type, and sometimes a value. Types can be primitive ones (e.g., string, Boolean and integer) or a reference to an already defined type of information.

An ITS consists of multiple *containers* and communication *channels* connecting them. Containers are computational nodes for *sensors*, *actuators*, *utilities*, and their controller *managers*. Sensors perceive *events* from the environment and use their methods to generate *notifications* to other components. These notifications can trigger the execution of methods, which in turn can generate new notifications. The triggered methods can belong to other sensors, actuators that act on the environment, or utilities that are pure software components working inside an ITS. Managers are agents that use the available information to check their goals and decide what tasks to attempt. The execution of these tasks can invoke methods.

Outside the ITS, there is an external *environment* that comprehends *things* (e.g., roads and signals). *Persons* actuate in this environment. Some of them can be in *vehicles*, either as drivers or passengers. All these elements are subclasses of a general *place* class and can interact with the others through their interfaces. A place contains multiple *spots*. These are both elements that can be observed and where *containers* can be located.

Following MDE practices, the ITSML is specified with a metamodel that includes the previous concepts. Metamodels define the abstract syntax of graph-based MLs, i.e., their primitives and constraints [19]. Meta-MLs (MMLs) to define these metamodels usually have as primitives *nodes*, *relationships*, and *roles* (i.e., the ends of relationships that connect them with nodes). *Properties* are attributes or adornments of these elements. Some examples are names, visibility, or cardinality indications for roles.

There are also some standard relationships included in many MDE MLs, which correspond to similar ones existing at the level of their MMLs [21]. Examples of them are inheritance and composition relationships, which the ITSML also includes. These relationships are introduced to facilitate making specifications with the MLs. Following usual conventions in the area [22], in diagrams below, relationships with triangles represent inheritance, and with diamonds represent aggregation, using filled diamonds for composition and hollow diamonds for plain aggregation.

Elements in these MLSs can be in packages, and their fully qualified name includes the package name. For instance, the package “ITS” for the ITSML, and the fully qualified name of “component” as “ITS::component”.

At the level of the ITSML, the language also includes mechanisms to instantiate classes. In the metamodel, for a *name* meta-type, there is another *Iname* to represent its instances. The *instanceOf* meta-relationship links both meta-types. This allows linking instances to their actual types in models. For instance, the *ISensor* meta-type is linked by the *instanceOf* meta-relationship to the *Sensor* meta-type. With this, a model could have a sensor type named “Temperature sensor” (i.e., *Sensor* stereotype) with an actual instance named “Sensor1” (i.e., *ISensor* stereotype).

### 2.2. Social Properties

The work on *social properties* [12] seeks to provide effective ways to apply the knowledge from Social Sciences in Software Engineering. This entails having guidelines for its application supported by software tools. Its theoretical background comes from the Activity Theory (AT) [23], which is focused on the description and analysis of social activities and their contexts. It establishes a conceptual framework and the rules that govern the evolution of activities.

The core concept of the AT is the *activity*. It represents a transformation act that is both intentional and social. The intentionality appears because all the participants endeavor to satisfy some high-level needs through the activity. The social dimension is due to the fact that every activity happens in a society/group, i.e., a subject (or group) carries it out using artefacts created by a society. Activities can mix both mental (e.g., learning and planning) and physical (e.g., driving or lifting a weight) aspects.

An *activity* is performed by *subjects* pursuing their *objectives*, which represent their needs. These objectives are satisfied by the activity’s *outcome*. The transformation of *objects* using *tools* produces those outcomes. The *community* represents the group where the activity takes place. In a wide sense, it represents the set of subjects that share an environment and meanings both now and historically. Two artefacts mediate the relationship between the activity and its community. The *division of labor* specifies how the community is organized for the execution of the activity. The *rules* are cultural artefacts that influence the activity, e.g., norms and tales. The ensemble of all the previous elements constitute the *activity system* of an activity.

There are two additional types of transformations. Activities are decomposed into *actions*. These are conscious intentional acts, but their goals do not correspond to high-level needs. Actions are in turn decomposed as *operations*, which are to a large extent non-conscious and only depend on the environment conditions.

Specifications of social contexts take the form of networks of activity systems linked by shared elements. These elements can play different roles in those activity systems, e.g., the object of an activity system can be a rule in another.

These networks evolve over time. Their elements change (e.g., they suffer damage or acquire new capabilities), and they appear and are destroyed. These changes produce competing goals and inadequacies between purposes and characteristics of elements. The AT names these conflicts *contradictions* [24]. Contradictions make activities somehow unsuitable to satisfy the subjects’ needs. For this reason, subjects try to remove or mitigate them by changing the activity systems. For instance, they can change some elements or introduce alternative activities. These changes are usually the origin of new tensions that produce further evolution.

The UML-AT ML [12] formalizes this theoretical framework with a Unified-Modelling Language (UML) profile [22]. It uses stereotypes to represent these concepts and their relationships. It also includes additional elements to facilitate the specifying tasks. For instance, the type *artefact* represents any type in an activity system, and the relationship *change of role* that a given concept adopts different types. It also includes some usual primitives in MDE MLs [21] such as relationships to represent aggregation and inheritance, and cardinality adornments.

In this context, *social properties* represent knowledge about social activities applicable in different contexts and extracted from literature in Social Sciences. Their specification uses a template that combines UML-AT diagrams and text.

A pattern-matching algorithm uses those diagrams to check properties. It looks in available information for instances of the query specifications. The appearance of an instance of a property is used to explain the meaning of the information located, and its absence that those specifications do not satisfy the property.

This approach has been used in a variety of domains [12]. Some examples are social analysis, cooperative work, and e-learning.

## 3. The SADAS Framework

The SADAS framework seeks to extend traffic systems with social information. To do so, it must interpret the available raw information to derive social information. For instance, from the observed position of a vehicle and a map indicating a nearby park, that there could be children, and thus speed must be reduced. This derived information includes characteristics of the people and their movements, activities, companion, and environment. From it, a SADAS can infer potential interactions with those elements in the near future and provide information based on that to the rest of the traffic system.

To achieve this functionality, the framework provides an ML to specify social information and its transformations (see Section 3.1). Traffic social properties are ready-to-use specifications that represent knowledge of recurrent situations or changes (see Section 3.2). A MAS architecture is used to process all these specifications (see Section 3.3).

### 3.1. SADAS-ML

The SADAS-ML is a specific language for managing social information. It defines primitives to represent that information (see Section 3.1.1) and its processing (see Section 3.1.2). For the information, it extends elements from the ITSML and UML-AT MLs (see Section 2) following the guidelines in [21,25]. For the processing, it mainly adopts the AT concept of activity [23] (which is already present in the UML-AT, see Section 2.2), and the new one of *transformation*. The language also supports some general features regarding type instantiation and the use of variables (see Section 3.1.3).

#### 3.1.1. Social Information

**Figure 1:**
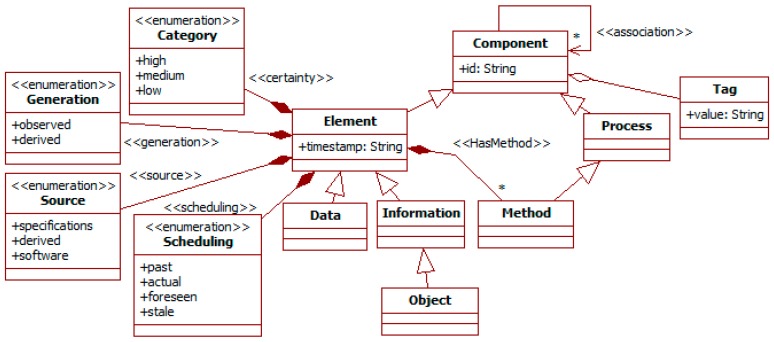
Partial SADAS-ML specification with its base primitives for social information. All the elements belong to the metamodel.

The specification of social information relies on a conceptual hierarchy whose base element is the *component* meta-type. Figure 1 summarizes the base primitives related to it. They are the more abstract types of the SADAS-ML.

The *component* meta-type extends the ITSML *component* (see Section 2.1) and represents any concept in the problem domain. Its only attribute is the *id*, which must be unique among all instances of a specification. It can also have an arbitrary set of string *tags* to describe it in a tailored way. For instance, to depict a point as a school or a museum. It can define arbitrary *associations* with other component meta-types.

The component sub-types in the metamodel are the *element* and the *process*. The former represents concepts, and the latter functionality.

The *element* extends the *component* meta-type and the UML-AT concept of *artefact* (see Section 2.2). It adds a *timestamp* attribute and may have *methods* (which extend the SADAS-ML *process* meta-type). The timestamp corresponds to the moment of its creation or update. It also includes reliability attributes to characterize the confidence in their information. These are the following. *Certainty* indicates how probable the information is to be true using a value in *category*. For instance, a sensor working in an environment with high-levels of noise probably perceives data of *low* certainty. *Generation* classifies information between *observed* and *derived*. For instance, the maximum speed of a vehicle is *observed* when asserted according to sensor data, while *derived* when deduced from observations on its previous speeds. In the case of observed information, the *source* identifies its generator (e.g., specifications, sensor, or software). *Scheduling* distinguishes between *past*, *actual*, *foreseen*, and *stale* information. Past information was applicable, actual is at the current moment, foreseen is expected to be in the future, and stale was foreseen but no longer and it did not become actual. For instance, when pedestrians are arriving at a crosswalk, it is foreseen that they will continue and cross; when they disappear from images, the expectation that their position is close to the last one will become stale after some time without them reappearing. These attributes are used to prioritize decisions based on information.

The element sub-types in the metamodel are *data* and *information*. The former is used to represent sensor observations as opposed to the latter for abstracted social information.

**Figure 2:**
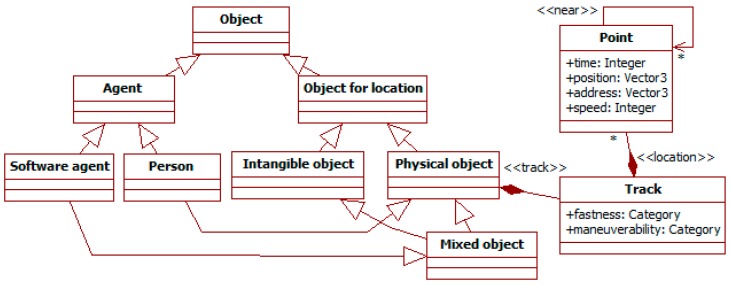
Partial SADAS-ML specification with the sub-types of the *object* meta-type.

Information has as its sub-type *object*, which represents elements from the traffic system or the external environment. Figure 2 shows it with its immediate sub-types.

The *object for location* meta-type distinguishes objects according to whether they have an associated location. They can be *physical* (e.g., a person, vehicle, or sensor), *intangible* (e.g., a group, norm or interaction), and *mixed* (e.g., an activity or a museum with physical facilities and an intangible website). Physical objects have a *track* to describe their movement. This has two attributes for its general characterization. *Maneuverability* indicates the observed ability to change direction sharply, and *fastness* describes the potential fastest speed. Both attributes take values in *category* (see Figure 1) to give a qualitative approximation to those characteristics. The track path is described as a sequence of *points* over time. The *near* relationship indicates that two points are in close positions.

The *agent* sub-type of object represents an intentional and social entity in the vein of the AT subject (see Section 2.2). The ML introduces it at this level to solve the dichotomy between its two sub-types: *software agents* in traffic systems, which are mixed objects; and *persons*, which are physical objects.

**Figure 3:**
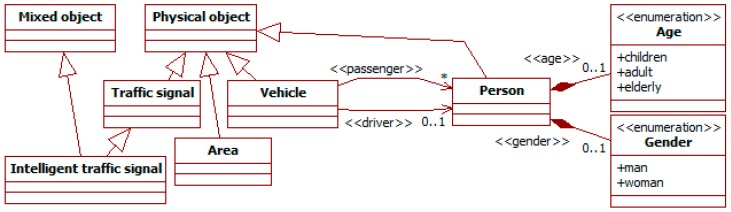
Partial SADAS-ML specification with the sub-types of the *physical object* meta-type.

Figure 3 shows the hierarchy of concepts starting in the *physical object* meta-type. There are several sub-types: *area*, *traffic signals*, *person* and *vehicle*.

*Areas* represent terrain extensions whose perimeter is delimited by the points in their track. They are used to specify delimited spaces, such as buildings or neighborhoods.

*Traffic signals* describe any sign or signal related to traffic. Examples of these are signals of speed limits or marks delimiting lanes. Its *intelligent traffic signal* sub-type is a mixed object. It includes as physical components traffic lights (with red, green, and yellow lights), a display, a speaker, and cameras to perceive surrounding traffic of both pedestrians and vehicles. It also includes the software controllers for these elements, which are intangible objects. With these features, this intelligent signal can represent most devices of this type in literature.

The *person* sub-type represents people in traffic. Its attributes describe their physical state (e.g., *age* and *gender*) or their socio-economic characteristics.

People can travel in vehicles represented by the *vehicle* meta-type. It has several sub-types not shown in the figure, such as *car*, *motorbike*, *bicycle* and *scooter*. Their characteristics are described with specific values for the attributes inherited from their super-classes (e.g., the fastness from physical object), or additional attributes. Persons can be their drivers or passengers, as in the ITSML (see Section 2.1).

**Figure 4:**
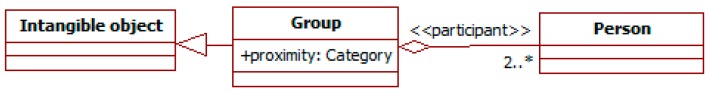
Partial SADAS-ML specification with the *group* and related meta-types.

People can also move together in *groups* (see Figure 4). These are intangible objects, as they only represent the association among their participants. A group has a *proximity* attribute to represent how close those ties are. For instance, a family group usually has a *high* value of proximity.

#### 3.1.2. Processing

**Figure 5:**
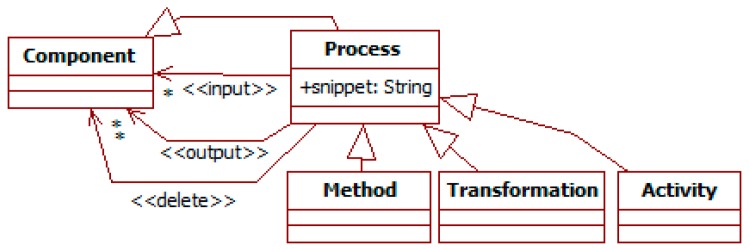
Partial SADAS-ML specification with the sub-types of the *process* meta-type.

As introduced before, the *process* meta-type represents functionality (see Figure 1). This functionality for a type is described as transformations of *component* types (see Figure 5). Its semantics is that, when instances of all its *input* types are available, it produces instances of all its *output* types, meeting the constraints specified in its definition. Outputs can also be marked as *delete* to indicate that an instance and all its dependents (i.e., targets of aggregation relationships) will be removed. Processes can have “snippets” to further specify these changes. Such snippets are described with arbitrary languages, e.g., plain text, code, or logics.

There are three sub-types of process (see Figure 5). *Transformations* represent automated derivations whose execution only depends on their information context. That is, when available information meets their input constraints, they always generate their output. *Methods* are attached to the element of which they represent a capability. *Activities* have an intentional and social meaning for their participants according to the AT [23]. They indicate specific roles of inputs and outputs (e.g., subject, rules, or tool), and always have at least one agent acting as the *subject* that executes them.

Inputs and outputs can have adornments. Existence adornments specify conditions on the availability of instances of a given input type: “NOT” for the absence of instances; “ANY” for the presence of at least one instance; “ALL” for every available instance of the input type. Both inputs and outputs can use cardinality adornments: * to indicate any number of instances equal to or greater than 0, a number for that exact number of instances, or a range N..M for several instances equal to or greater than N and equal to or less than M.

#### 3.1.3. General Characteristics

Social information is specified using these meta-types (e.g., *person* or *vehicle*) in models. As in the ITSML, for every *name* meta-type, there is an *Iname* meta-type for its instances, and an *instanceOf* meta-relationship to link them. This allows distinctions to be made in models between types (with the stereotype *name*) and instances of these types (with the stereotype *Iname*). For instance, *Person* is the stereotype of instances of the meta-type *Person* in models, which are types (e.g., *Young driver*); the instances of these types use the stereotype *IPerson* (e.g., *Person1* who is a young driver). The actual type of a model instance can be indicated with the *instanceOf* relationship. In the following diagrams, when the type of an *Iname* instance is omitted (no specific *instanceOf* relationship), it is considered to belong to any type of the indicated *name* meta-type.

Attributes of both types and instances can have values or not. Values introduce constraints over attributes. For instance, a *person* type without a value for *age* represents instances of any age, but with a value, only instances of that specific age. If the attribute has a value, this can be a constant or a variable. Constants are represented with their literal (e.g., 5, true or “museum”). Variables are represented with a name starting with an underscore (e.g., *_AGE*). Variables act as placeholders for the actual value of the related attributes. All the appearances of a variable in the same diagram take the same value. For example, this can be used to indicate that several instances of person are the same age.

A SADAS uses observations from sensors as inputs to describe its environment using the previous concepts. It categorizes surrounding objects and assigns attribute values to them. For instance, a person walking alone with a speed below the average (i.e., *fastness low*) may suggest an elderly person (i.e., *age elderly*) or somebody with mobility impairments [26]. The latter information is not actually observed, but derived using social knowledge

### 3.2. Traffic Social Properties

*Traffic social properties* describe prototypical social contexts or processes over them. For instance, on the behavior of pedestrians in groups [8], passengers in vehicles [10], or AT contradictions (see Section 2.2). They follow the template for general social properties [12] but using the SADAS-ML.

**Figure 6:**
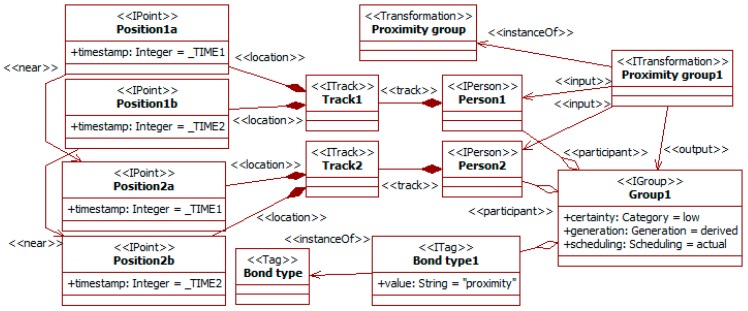
Property to derive information about social bonds from position information.

Figure 6 shows an example of property. It considers that people moving together for a certain time probably belong to some kind of group [27]. The diagram uses type instances to consider actual data available in the SADAS.

The diagram shows two instances of person (i.e., the *person1* and *person2*) that are the input of the *proximity group1* transformation. These instances have tracks with several positions. The diagram uses variables to indicate which positions are simultaneous. For instance, *position1a* and *position2a* happen at the same time, as they have the same value for the attribute timestamp (the shared variable *_TIME1*). The *near* relationship indicates that the linked positions are close in the space. The fact that *_TIME1* and *_TIME2* must be different but close in time cannot be graphically indicated. The snippet of the transformation can be used to specify it.

When the available information meets the previous constraints, the transformation creates an instance of the *group1* group type that includes as participants the two instances of person. The instance of the *bond type* tag type highlights that the group is derived from the observation of the proximity in movements. This also leads to a *low* value for the *certainty* attribute of *group1*.

### 3.3. Architecture

The SADAS architecture includes components to manage social information as specifications compliant with the SADAS-ML (see Section 3.2), and to integrate those components with others of traffic systems. The information processing follows an agent-based architecture for knowledge-based systems using external resources called MASSA [28]. It decouples the pipeline to preprocess information from the knowledge-oriented components. To facilitate reasoning, a SADAS uses a shared representation of the context in a *social engine*. Agents can consult and modify it using resources that implement the observer pattern. This follows common practices in context-aware systems, particularly the FAERIE component architecture [14].

**Figure 7:**
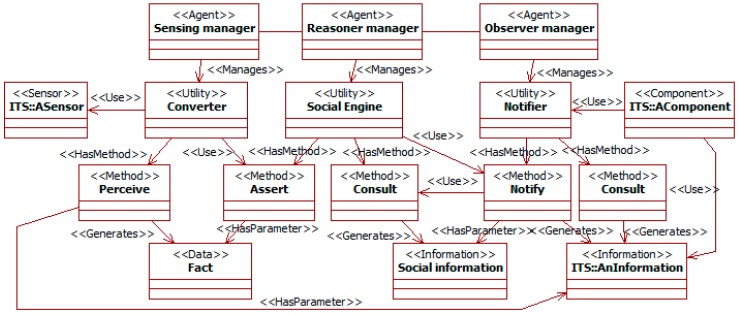
Components in the SADAS architecture. Concepts belong to the ITSML [11]. Components from package “ITS” correspond to standard traffic systems outside a SADAS. Only the key relationships are depicted.

Figure 7 shows the main components of the architecture. It uses concepts of the ITSML such as agent, utility, or sensor (see Section 2.1). The components *ASensor* and *AComponent* represent elements from the traffic system where the SADAS is integrated. The other components are specific to this study and work internally with SADAS-ML specifications.

There are three types of agent. *Sensing managers* get observations from the traffic system external to the SADAS. *Reasoner managers* manipulate the SADAS-ML information. *Observer managers* communicate the relevant information to the external system. These agents use several support utilities in these tasks.

The utility *converter* of sensing managers acts as an observer for the external components. These external components can be, for instance, sensors and other SADASs. The *perceive* method captures the events produced by those components and notifies them to the relevant components.

The *social engine* utility of reasoner managers implements a rule-based engine. Its Knowledge Base (KB) contains facts and traffic social properties. The engine provides two methods. The *assert* method allows new facts to be introduced in its KB. The *consult* method supports queries to the KB.

The *notifier* utility of observer managers supports the communication of social information to external components. It implements the *notify* method to be reported on changes in the KB. This utility also implements the observed element of the observer pattern. In this way, external components can use it to be notified on changes in the KB. The *consult* method supports external queries to the KB.

The workflow is as follows considering only utilities. When there is an external event, the *converter* is notified about it. It translates that external event into facts as SADAS-ML *data* instances and inserts them into the KB. When it has finished inserting new information, it triggers the engine reasoning to derive information and check properties. When this process ends, the engine notifies that to the utility *notifier*. The notifier uses the engine method *consult* to read the KB and gets the relevant information as SADAS-ML facts. It translates these facts into suitable formats for the rest of the traffic system. Then, the notifier changes its state, and the external components (e.g., actuators and other SADASs) acting as observers get notified on it.

When the previous process requires complex reasoning (e.g., translations using ontologies or requests among agents with speech acts), manager agents are introduced in the workflow. In that case, communications between utilities are mediated by their managers, so these can perform their tasks.

This design enables flexible management of social information. It isolates knowledge-related components in the SADAS, while components that participate in observer patterns (i.e., converters and notifiers) provide the interface with the rest of the traffic system. These same components and their managers carry out the translations among the external information and SADAS-ML specifications. The translation-related components can be reused among different SADASs, as they are tailored to specific targets (e.g., certain types of sensors). Inside the SADAS, the rule-based approach of the social engine and the reasoner manager facilitates modifying the used knowledge just changing the included traffic social properties.

## 4. Experimentation

This section illustrates the application of the previous framework (see Section 3). It includes a case study (see Section 4.1), and the discussion on it and additional experimentation (see Section 4.2).

### 4.1. Case Study: Intelligent Traffic Signals with Social-Awareness

The case study considers a commercial area with shared spaces. There, several systems act together to make traffic flows safe and fluid using different signaling systems. The introduction of a SADAS is intended to use social information in this context. It will help to better describe the environment and profile people according to their characteristics, means of transport, and activities. In turn, this will be used to adjust traffic signal behavior and send tailored warnings to cars.

The traffic systems in the area are two presented in literature. First, there is an adaptive control of traffic signals [15]. It is a hierarchical system that makes decisions at several levels: the area, intersections, and specific crosswalks. This control relies on several sensors to monitor the surrounding traffic, including cameras and inductive loops. The second system is a distributed DAS for the detection and tracking of pedestrians using car cameras in Vehicle Ad-hoc NETworks (VANETs) [16]. The system supports cars to exchange information about their detections to reduce problems such as pedestrian occlusion. In both cases, the analysis of images from cameras enables extracting of different characteristics of traffic and their participants, e.g., pedestrian location, type of vehicle, obstacle speed, person age and gender [17,18]. The reliability of this information varies according to capture conditions.

The next sections discuss the previous aspects. Section 4.1.1 focuses on properties to characterize people and the environment in this problem, and Section 4.1.2 on properties that use the social information to make decisions on traffic control. Section 4.1.3 discusses some specific aspects of the architecture of this SADAS. Finally, Section 4.1.4 provides some examples of how the SADAS uses the previous elements.

#### 4.1.1. Context Characterization

The SADAS works here with social information about four main groups of elements: the area, which is a commercial one; the used vehicles; people’ characteristics and activities; and companion. These groups of information are considered mutually interacting rather than isolated. Next, some examples of traffic social properties for these groups are introduced.

**Figure 8:**
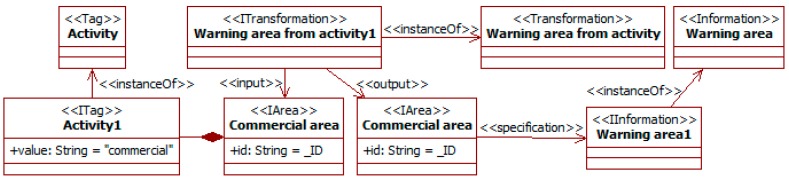
Property to identify warning areas of special attention from their activity.

The type of area and its facilities are known to change some people’ attitudes (see for instance [29,30]). For example, people walking in a leisure area (e.g., a park, cultural facilities, or commercial area) are more likely to wander slowly around. There are also areas where vehicle drivers must be particularly aware of vulnerable pedestrians, such as children near a park or school, people with limitations close to a hospital, or distracted people in leisure areas. Figure 8 considers this with a traffic social property that identifies some of these areas of particular concern.

The *warning area from activity* transformation looks for areas tagged with instances of the *activity* tag type. These indicate specific uses, in this case “commercial”. When one is found, an instance of information of the *warning area* type is created and attached to the area. This instance could contain information to operate in that area, e.g., maximum speed or types of vehicle allowed. The use of the variable *_ID* indicates that the two instances of area are the same, as they have the same identifier. This allows modifications of instances to be specified.

**Figure 9:**
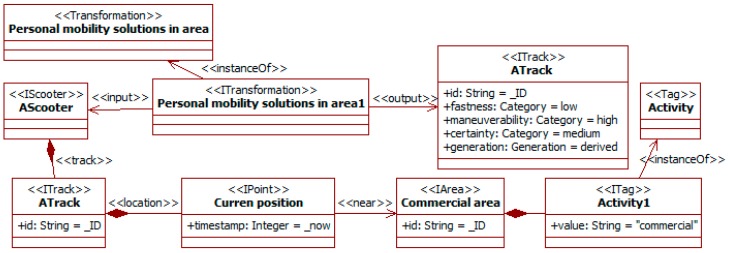
Property about movement characteristics from the area activity and the type of vehicle.

The type of vehicle used also implies differences in traffic behavior (e.g., for pedestrians [8], cars [10], bicycles [9] and scooters [31]). Vehicles have different characteristics (e.g., maneuverability, maximum speed, applicable norms, and allowed lanes). They are also used in different ways (e.g., scooters driven by children or adults), frequently depending on the purpose (e.g., a leisure walk or last-mile travel). One way to draw some expectations about this purpose is using the area activity. For instance, in leisure places (e.g., cultural and commercial areas), people using personal mobility solutions (e.g., bicycles and scooters) are more likely to be taking a leisurely walk than moving in transit to some place. For this last purpose, these areas are not the best option, as they are usually crowded. Figure 9 depicts a traffic social property that considers this knowledge.

The transformation *personal mobility solutions in area* considers *scooters*, which are a sub-type of *vehicle*. When they are moving in a leisure area (here a commercial one), the purpose of their drivers is drawn as leisure. The *current position* of the scooter is extracted from its track (i.e., the *ATrack*). The variable *_now* is a special one in the SADAS-ML that means the actual moment. The commercial area is characterized using tags (as in Figure 8). The presence of the scooter in it is represented using the *near* relationship. The pleasant goal of the travel activity affects the way of moving described in the resulting *ATrack* for the scooter. It has a *low fastness* related to its wandering nature, though keeping a *high maneuverability* (i.e., the probability of sharp changes in direction) related to the inherent characteristics of the type of vehicle. This last characteristic is part of the definition of the scooter type.

The previous transformation derives information for the track. It indicates that with the value *derived* in the *generation* attribute. The *certainty* about this derivation is *medium*.

**Figure 10:**
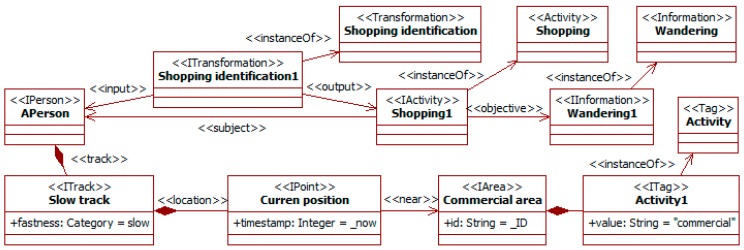
Property about pedestrian’s activity from movement characteristics.

People’s individual characteristics and activities also affect their behaviors in relation to traffic. For instance, young drivers are more prone to risky behavior and elderly ones with limited capabilities to suffer accidents [31], and the gender and the moment of the week establish different preferred places to go [30]. This knowledge can be used to extract information on expected behaviors, characteristics, or state. Figure 10 illustrates this possibility with a traffic social property that makes a guess on the kind of activity a pedestrian is engaged in from her/his observed movement.

The transformation *shopping identification* considers a pedestrian *APerson* moving slowly in a commercial area. From this information, it derives that the subject is probably performing an activity of type *shopping* with the only objective of *wandering*. Certainty attributes are omitted in the figure.

**Figure 11:**
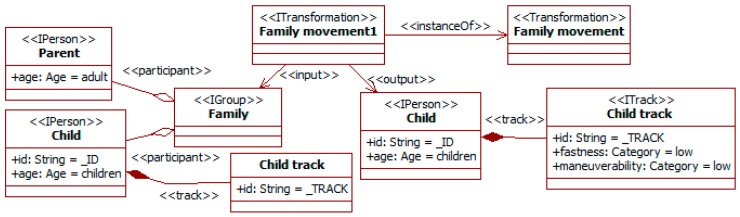
Property about child movement with an adult.

**Figure 12:**
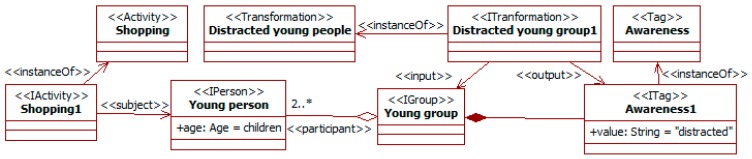
Property about attention level in groups of young people.

The last group of social information considered is related to social bonds among participants. Companion affects perception and behavior in traffic. For instance, passengers usually reduce accident risk, but less so for young drivers [10]. This kind of traffic social property can also be considered. Figure 11 shows that the movement of a person can be changed by the companion, here that of children by the presence of adults, and Figure 12 that companion can alter the attention level to traffic.

The transformation *family movement* takes as input an already identified group with a *child* and the *parent* (i.e., really an accompanying adult). This can be the result of a previous property (see Figure 6) and feature extraction from images. In this situation, children usually have quieter movements than when they are alone, as there are adults surveying them. This is represented by modifying the *child track* with a low value for the attributes *fastness* and *maneuverability*.

The transformation *distracted young group* considers that groups of young people involved in a leisure activity (here *shopping*) probably pay reduced attention to the surrounding traffic. This awareness is characterized with the specific tag for this aspect attached to the group.

The previous traffic social properties are used to derive information about the environment and the expected behavior of participants. The SADAS uses this to assist users and get a safer and more fluid traffic.

#### 4.1.2. Traffic Decisions

The characteristics of people and the environment influence how traffic systems act, and social information contributes to this characterization. The SADAS helps here to consider it in the original systems. The next properties illustrate this point.

**Figure 13:**
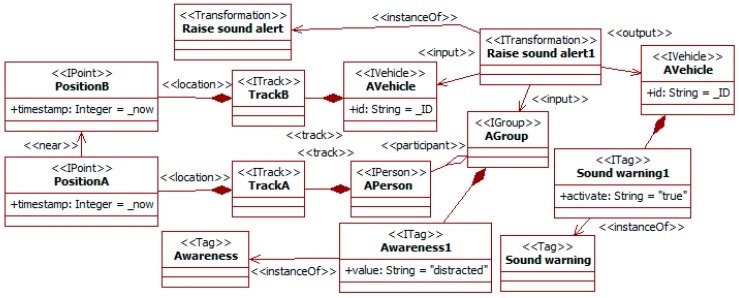
Property about triggering sound alerts in the vicinity of distracted groups.

Social information can be used to choose when to activate the safety measures in the car detection system of [16]. Some of the previous properties are related to the attention level of pedestrians (see Figure 12). Moreover, the growing presence of silent vehicles (e.g., bicycles and electric cars and scooters) increases the probability of their reaching pedestrians’ positions unnoticed [32]. Figure 13 considers this kind of situation.

The *raise sound alert* transformation looks for vehicles (i.e., the *AVehicle* instance) close to pedestrian groups with potentially low levels of attention (i.e., the *AGroup* instance). This level of attention is characterized with the tag of type *awareness* (as in Figure 12). The fact that the vehicle and the group are close is described using the *near* relationship with the current positions of the vehicle and some group participant. The suggestion of activating the sound alert is characterized as another tag of type *sound warning* linked to the vehicle.

**Figure 14:**
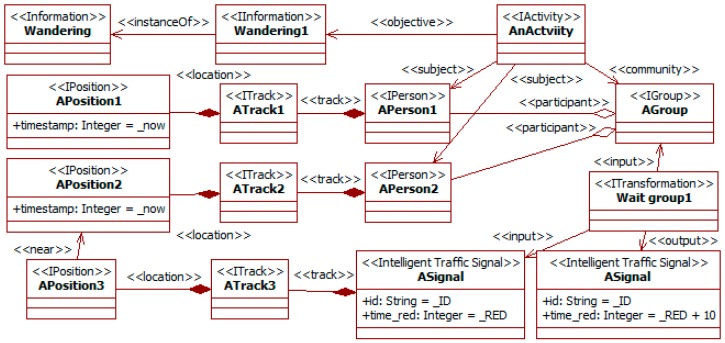
Property about traffic lights to reorganize groups. The transformation type is omitted in the figure.

The social information can also be used in the ITS that controls traffic lights [15]. For instance, to accommodate the duration of those lights to the presence of pedestrian groups. A group involved in a wandering activity (e.g., shopping or visiting a place) is likely to become dispersed. When such a group arrives at the traffic lights, some of their members may cross while others wait. This situation frequently causes some pedestrians to walk when their light is red. This danger can be reduced if all the group members remain on the same side of the road. To do that, the duration of the red light can be increased to force regrouping. Figure 14 works on this situation.

It considers a group (i.e., the *AGroup* instance) performing a wandering activity (i.e., the *AnActivty* instance) with an objective *wandering* (as in Figure 10). This group could also be characterized using a tag *awareness* (as in Figure 12). The fact that this group is dispersed is described from the positions of its members (i.e., the *APerson1* and *APerson2* instances). At least two members have positions separated by a distance over a certain threshold. This condition is part of the snippet of the transformation *wait group1*. Simultaneously, some members of the group (here *APerson2*) have reached the position of the traffic lights (i.e., the *ASignal* instance). This is characterized with the *near* relationship. When available information meets these conditions, the transformation increases the time for the red light (i.e., the attribute *time_red*).

#### 4.1.3. Architecture

The architecture of this SADAS is very close to the generic one (see Section 3.3). There are two groups of components: those from the original systems [15,16]; and those of the SADAS. As in Section 3.3, the component types of the architecture come from the ITSML (see Section 2.1).

The original components are represented by *sensors*, *actuators* and *utilities*. Managers are not included, as agents are more complex components and they are not required in these traffic systems. The only common requirement for the considered components is that they offer a public interface with consult methods. If they produce notifications, they must implement the observed component of the observer pattern. Components whose behavior the SADAS changes must also provide modify methods. In most cases, information for the SADAS comes from utilities that perform some processing of the raw data, such as feature extraction from images.

The car detection system [16] can provide information on obstacle detection on elements such as cars and pedestrians, and thus about their positions over time. The control system for traffic signals [15] includes cameras that contribute to this tracking. It also provides the position of its signals and crosswalks. Information about areas can be extracted from publicly available sources, such as open data initiatives from city governments.

The SADAS just includes the original components of its architecture (see Figure 7). Agents are not required here, as there are not complex processes beyond the utilities. Specific converters and notifiers are required to communicate with the original components (those for the systems in [15,16]). The rest of the SADAS components are the general ones and do not need to be modified. Only the contents in the KB of the social engine change to be adapted to the current context. Nevertheless, parts of these contents can be reused from other systems (e.g., some properties, facts, and information types), as they are independent of specific external devices and utilities, and applicable to multiple social contexts.

The previous information allows more precise expectations on people’s behavior and movements to be derived than in the original systems. For instance, considering the probable influence of companion or the characteristics of people and vehicles. Attributes such as the expected *maneuverability* and *fastness* of an obstacle can help to determine a safety area around it with the potential next positions. It can also help to identify the most suitable warning mechanisms in a situation from people’s awareness and capabilities. The social information can also be an aspect to consider adjusting traffic flows. For instance, the times of traffic lights can be dynamically adapted to the presence of groups or people with mobility issues.

#### 4.1.4. Functioning

An example of typical workflow of this SADA might be the adaptation of traffic lights and alarms to a group of friends doing shopping. It starts with information from the original DAS [15] and ITS [16], goes to the SADAS that derives new information, and sends part of this back to the actuators in the original systems.

Initially, some ITS utilities can consult (or be initialized with) information about the area. SADAS notifiers receive this information and assert it as *area* instances with the *activity* tag with value “commercial” (as in Figure 8).

When the traffic systems are working, their cameras detect persons and vehicles, and start tracking them. Notifiers again receive this information. They respectively assert it as *person* and *vehicle* instances with their related *track* instances (as in Figure 6). This tracking continues over time, adding to those tracks new locations as *point* instances.

For the group of friends, the previous points are close over a period. When the reasoning cycle of the social engine starts with this information, the property about social bonds (see Figure 6) becomes executable. The pattern-matching algorithm finds that the inputs of the transformations are available and meet the constraints. Thus, the engine triggers the *proximity group* transformation and generates the *group* instance among the previous *person* instances.

The information about the area and the people’s tracks also allows the property about activities to be triggered (see Figure 10). The *track* instances of friends indicate a slow movement, i.e., with *low fastness*. At the same time, their locations are close to the area (in fact inside). This is asserted by a transformation that compares positions in its snippet. From this information, the property derives that these *person* instances are engaged in a *shopping* activity with a *wandering* objective. In turn, this information with a group of youngsters would cause the property about the attention level (see Figure 12) to derive that the group of friends is probably moving in a distracted way. This last information corresponds to an instance of the *awareness* tag type.

When the positions of one of these friends and a vehicle become close, the DAS should trigger a suitable alarm. The property on sound alarms does it (see Figure 13). The reasoning on positions is similar to the one for the previous group property. The group of the person also has an instance of the *awareness* tag with the *distracted* value attached. Thus, the inputs of the property transformation are available, and it generates the outputs. Here, the output is a tag of the *sound warning* type attached to the vehicle. This information is sent to a SADAS converter that is observed by external components. Thus, its state change triggers the sound alarm of an external observing actuator in the DAS.

The previous information can also change the duration of traffic lights in the ITS. In this case, the property (see Figure 14) requires that the positions of some group members are not close, and some of them are close to the crosswalk with the lights. The information on people’ positions could come from the cameras in the ITS or the DAS. In this last case, integration could be achieved through the direct exchange of information between the SADAS in both systems, as far as suitable converters and notifiers were configured for it. The previous information makes the property executable, and its triggering modifies the lights’ duration.

### 4.2. Discussion

The SADAS framework has been tested in several experiments. These considered different problem domains and research studies. Regarding the ML, they assessed its suitability to describe different types of participants, vehicles, and traffic systems, as well as transformations of this information. To evaluate the impact of using this information in traffic systems, experiments carried out simulations using the framework for ITS (see Section 2.1) as basis. This enabled it to be validated that SADASs could be included in those traffic systems, and that social information changed their behavior and actually improved traffic. This section discusses the main findings of these experiments.

Regarding the modelling of participants, experiments have focused on profiles according to literature. These included differences regarding age [26,33,34], gender [26,34], culture [35], and socio-cultural level [36]. In general, these studies offer statistical analyses of the relation between some profile characteristics and behaviors in traffic activities. These can be translated to the SADAS-ML using sub-types of the *person* meta-type that include the required new attributes and their values. Their effect is modelled through processes, which use those characteristics as inputs to affect the values of other information.

In the case of studies that involve the actual decision-making of participants (e.g., [29,37,38]), the SADAS-ML has some limitations. It allows modelling when an agent chooses an activity because it achieves some of its objectives. However, it does not include primitives to reason about those objectives (e.g., preferences, decomposition, suitability, or hard and soft goals). That kind of primitives has already been addressed in other studies. For instance, literature in the agent paradigm has paid attention to goal and means-ends modelling and analysis [13]. Some of these primitives appear in the base languages of the SADAS-ML, i.e., the UML-AT and the ITSML (see Section 2). However, these only address goal decomposition and positive and negative contributions to goals. Such primitives are not currently part of the SADAS-ML.

Different types of vehicle [9,31,32,39] can be modelled as sub-types with specific characteristics of the *vehicle* meta-type. These vehicles can embark systems and people.

The main limitation found regarding the modelling of physical objects (e.g., people and other elements in the environment) has to do with their borders and movements. This is relevant for instance when dealing with collision detection. An approach similar to that for areas (i.e., defining their perimeter with points), could partially solve the problem of borders. Nevertheless, reasoning with physical models is not the goal of SADASs. The relevant information should be provided by external components related to those aspects. Regarding movement, the description of tracks as sequences of points causes a quick growth of data. A solution to reduce it may be by adding new points only when the trajectory changes its direction or speed.

The modelling of traffic systems has been studied with smart roads [40], DASs [41], and traffic control [18,42]. In most cases, SADASs only need to know the information these systems provide or use through standardized interfaces (i.e., implementations of the observer pattern). When there is a need to detail the components of these traffic systems, they can be included through sub-typing of the *object* meta-type. The intelligent traffic signal is an example (see Figure 3), with attributes and methods for sensors (e.g., camera) and actuators (e.g., lights). This supports a high-level perspective of traffic systems, which is well-suited for SADASs as they focus on social information.

Describing the evolution of systems over time also requires specific primitives. This aspect is currently addressed with the attributes *timestamp* and *scheduling* in the *element* meta-type (see Figure 1), and indirectly through the relationships produce-consume (i.e., output-input) of information in the *process* meta-type (see Figure 5). This makes frequent using the snippets to reason about this dimension with conditions on the timestamp attributes. Additional primitives for qualitative reasoning on time could help to alleviate this issue.

As happens in the case study (see Section 4.1) and with time, numerical relationships among elements cannot be graphically represented in the SADAS-ML. Their specification resorts to snippets in arbitrary languages, where experts can use for instance their usual equations. Depending on their language, these snippets can be processed automatically (e.g., code). Even when this is not possible (e.g., free textual descriptions), these snippets are still useful to specify the system.

The experiments also enabled us to obtain an initial assessment of the impact of SADASs in traffic. They allowed information to be gathered on social aspects, and this changed the behavior of systems and participants in simulations. The results were similar to those reported in the case study. Regarding the impact of these changes in traffic flows, results were mixed. The considered social properties made systems very cautious with potential traffic problems (e.g., changes in obstacle trajectories). This was expected, as those properties were focused on safety issues. Though this reduced potential collisions, it also caused congestions in some flows and too many warnings on systems. Introducing some properties targeted to make flows more fluid and considering only collisions of high and medium probability could help to address these issues. The main conclusion is that the set of social properties must be carefully designed to get the desired balance between the different traffic aspects.

The final question raised from the experiments has to do with the need for guidelines to model and manage information. Beyond their description, there are no indications on how to choose when several concepts or relationships are possible (e.g., a mixed object as opposed to a physical object connected to intangible objects). It could also be difficult to design inheritance hierarchies for new needs, as choices entail tradeoffs that have impact on further extensions and the specification of traffic social properties. For instance, introducing new relationships to reason about time and decision-making. Choosing the right social properties for a SADAS is also challenging. Designers must consider the system requirements and problem tradeoffs. Experiments need to be crystallized in guidelines that help to understand the key aspects for decisions.

## 5. Qualitative Comparative Analysis

The SADAS approach is related to several fields of research. First, studies on characteristics of people and the environment that affect traffic (see Section 5.1). Second, research on the representation and management of the previous information to get new information (see Section 5.2). Third, the use of this information in traffic systems such as ITSs and DASs (see Section 5.3).

### 5.1. Characteristics of People and the Environment

Traffic is a complex phenomenon, with a variety of aspects that affect it. Literature usually groups them as related to individual persons, the companion in the movement, their means of transport, and the physical environment [20].

Individual characteristics are those depending only on the person and her/his state. It is common to disaggregate studies on traffic behavior regarding age [26,33], gender [26], and socio-economic level [36]. The actual state of the person can also have an impact on their traffic behavior, for instance regarding drowsiness [43] and stress [44]. These characteristics affect aspects such as risk perception and risk taking [26,33], response time [43,45], moving speed as pedestrians [38] and attitudes towards traffic regulations [34].

Usually, there are multiple participants in traffic. The previous individual characteristics can have distinctive effects on people’s movement and attitudes when considering them at the level of groups. For instance, age and gender in groups related to attitudes towards risk [33]. There are also specific characteristics linked to moving in groups. For instance, the level of space occupancy, which can affect the arrangement of groups travelling together in that space [27].

Vehicles have a direct impact on some movement characteristics such as maximum speed and maneuverability. They also affect people’s behavior, for instance in terms of risk assessment and speeding [36,39]. Their characteristics have also been used as clues on some traits of their drivers and passengers. For instance, the work in [36] about the choice of means of transport and socio-economic level, and the analyses of the perception of shared spaces in [5] and decision-making in [37].

The physical environment has also proved to have an impact on traffic aspects. It includes the physical space (e.g., lanes, road surface, street trees, and signals) [38], its conditions (e.g., weather, light, and road condition) [46], and type of space (e.g., shared, urban segregated or highway, and type of space activity) [4,30]. Again, this affects some movement characteristics (e.g., aqua-planning and visibility) and people’s attitudes [46].

The SADAS-ML supports the graphical modelling of most of these characteristics. For some of them, there are specific primitives that can be used without further changes (e.g., the relationship type between a vehicle and its driver). Others can be modelled using instances of general concepts such as the *information* or *tag* types with specific values in their attributes. In both cases, the ML can be extended through sub-typing, as done in the case study (see Section 4.1). For example, by creating a new sub-type of vehicle to represent roller skates. The manipulation of this information depends on the specific processes considered. Nevertheless, and as mentioned in the discussion of the experimentation (see Section 4.2), some elements and processes considered in these studies cannot be graphically modelled in the SADAS-ML. Examples of them are decision-making and reasoning on time aspects. These need to be addressed in the snippets of processes.

### 5.2. Management of Social Information

Traffic studies are undertaken in a wide range of disciplines, including Social Sciences (e.g., Urbanism, Sociology and Anthropology), Life Sciences (e.g., Health, Cognitive Psychology and Ecology), and Engineering (e.g., car and DAS development). Each of them has different methods of study and therefore of using information. In most cases, the management of this information is the task of human experts, who rely on natural language and analytical tools. When engineering automated systems, that information must be formally specified, so systems can use it properly.

Mathematical models have been used extensively in the area. They are particularly well-suited to model participants’ flows and motion [47], and when the focus is on specific calculations and algorithms, as in computational vision [17]. They are not so frequent when considering social information, as management here is usually more qualitative. In this case, available studies mainly focus on the development of models of participants’ behaviors at a low level of abstraction. For instance, inferring the response time according to physiological characteristics and processes [48].

Logics have also been considered regarding the modelling of social aspects. In particular, fuzzy approaches have shown their utility in dealing with qualitative reasoning on them. For instance, on modelling the crossing behavior of pedestrians from their critical gap [49] or of cyclists from their social force [50]. Nevertheless, and as with many mathematical models, these approaches have focused on specific decisions that depend on functions and thresholds over certain inputs. This makes it difficult to scale them up to the qualitative management of information, where decision-making is only the final step of several linked steps of reasoning, as shown in the case study.

Other general-purpose representations of information have also been used. For instance, rules to adjust image processing [51] or control traffic signals [42], and key-value tuples for context-aware systems [14]. These offer high flexibility to represent heterogeneous information, but lack mechanisms to represent complex information and specific methods for its management. Thus, these must be developed according to problem needs, which may require significant expertise and effort.

Looking for reasoning at a higher level of abstraction, some research has introduced the use of ontologies. These are formal and explicit conceptualizations of shared knowledge. Description logics facilitate reasoning over them. They have been used to derive new information and understand traffic situations [52,53]. Though ontologies offer the desired reasoning capabilities for SADASs, their usability by non-experts presents some problems [54]. Their support to create tailored modelling and examination tools is more limited than for other approaches. Moreover, adding assertions through logics requires a good understanding of the formalism and the underlying structure of the ontology.

Graphical MLs have also been applied, mainly related to simulations. The level of abstraction and scope vary among studies. There are developments of languages for specific aspects. For instance, ref. [55] allows specifying maps and vehicle speeds and paths, and [56] maps and participants in Smart Cities. Other studies adopt a wider scope regarding traffic aspects. For instance, the ITSML [11] endeavors to cover the specification of ITSs and their environment for different problems. It has been used in case studies using simulations of infrastructures such as smart roads [57], and in the context of MDE for integration with other types of systems [25]. Extension mechanisms for the MLs are considered in some studies, for instance in [25,56] through metamodel modification.

The SADAS approach is aligned with this latter group of works in the use of MLs defined through metamodels to specify information. Nevertheless, the SADAS-ML has several distinctive features.

Regarding its focus, the SADAS-ML is linked to general social aspects in traffic. This is something not considered in those previous studies or only for some specific aspects (e.g., age and existence of companion in [25]). The base of the ML in the AT [23] provides a suitable theoretical framework for this modelling. The SADAS-ML already includes elements to describe activity systems, but there are still AT concepts it does not include. Examples of these are the different decomposition levels of objectives and activities (see Section 2.2). When compared to the other AT-based language, the UML-AT [12], there are some shared concepts (e.g., the activity system, though modelled with different primitives). Others only appear in one language. Some from the UML-AT, such as contribution relationships, could be added to the SADAS-ML to address some of its shortcomings (see Section 4.2).

The SADAS-ML also differs from previous studies in that it incorporates the means to manipulate its information through the process meta-type and its related types. These not only address changes in information but also have a meaning grounded in the AT. This facilitates the specification of transformations extracted from social knowledge.

### 5.3. Traffic Systems

All the previously reviewed aspects about traffic participants and their environment exert an influence on this phenomenon. However, traffic systems such as ITSs and DASs only make limited use of them [6,7]. Some reasons are the difficulties in integrating heterogeneous information, dealing with uncertainty, and avoiding participants’ cognitive overload [41].

Available systems mostly use information about the driving area (e.g., type of road and lanes), environmental conditions (e.g., rain and light), and surrounding obstacles (i.e., their position, speed, and contour). Social aspects appear in some studies that can be grouped into two main domains. On the one hand, when considering problems affecting traffic in wider areas. Examples of these are incident detection, traffic forecasting, and demand estimation from timetables and area activities [58]. On the other hand, studies on users’ individual performance. The studies on drowsiness [43] and stress [44] detection using sensors belong to this group.

That information comes from low-level sensor processing in the case of nearby elements. For instance, multiple vision and radar techniques to track obstacles [17], or physiological sensors to detect stress [44]. For information on more distant elements or a wider perspective, systems resort to external sources. For instance, web services for maps and traffic in the area. There are studies on the abstraction and fusion of information to support a more complex management [58]. Nevertheless, abstraction is limited, for instance, characterizing participants as bounding boxes with certain speed as in [16].

The use of this information usually happens at a low level of abstraction. For instance, to track lanes or warn about potential collisions. It is highly dependent on the specific algorithms considered. For instance, systems based on artificial vision use raw images [17]. The development of integrated multi-purpose models is still an ongoing issue. Integration happens usually at the component level, for instance through notify-observe patterns [11], and less frequently with shared representations [59].

Being focused on social aspects, a SADAS uses the information provided by other components in traffic systems. Its converters and notifiers isolate the inner components from the external level of abstraction. They are responsible for representing that information as suitable SADAS-ML facts. In the reasoning layer, specific social properties can perform the required transformations on information, e.g., to abstract, integrate, interpret, or ground it. The case study (see Section 4.1) includes examples of these. This allows most of the types of transformation that appear in the studies reviewed to be made, though within the scope of social information as described here. Manipulations that require calculations must be specified in the snippets.

## 6. Conclusions

This paper has presented the SADAS framework for developing traffic systems (i.e., ITSs and DASs) that use social information. This information is related to characteristics of participants linked to their environment, activities and bonds, and their mutual influence. Considering this information allows extended models of traffic settings to be built, which results in better-informed decisions. Current traffic systems only partially consider this information, either at the macroscopic (e.g., to anticipate road load or conflicting points) or microscopic level (e.g., to measure response times or detect obstacles).

The paper includes an ML, an information management process, and an architecture. It is built on proven research in the areas of social information management [12] and ITS development [11].

The SADAS-ML offers an integrated conceptual basis to specify social information on traffic. It includes primitives to describe contexts and activities based on the AT [23,24] from Social Sciences. It can also specify traffic aspects including persons, vehicles, and their environment. The link between them allows traffic activities located in time, space, and social context to be described. The language also supports describing changes in information through processes (i.e., activities, transformations, and methods). These are specified with graphical primitives and snippets in arbitrary languages.

The language also provides extension mechanisms to tailor it. Among them are inheritance and instantiation of types in models.

This ML is used to describe the actual information of a system and prototypical knowledge applicable to it. The latter is specified as *traffic social properties*, following the template for *social properties* [12] but using the SADAS-ML. This knowledge is the basis for reasoning about social information. Its modularization as independent properties enables its flexible management, as modifications only require changing the properties considered.

The architecture that supports the processing of social information is based on the principles of modularity and flexibility. The overall design follows the MAS organization in [28]. It considers a pipeline of external tools. These provide information and methods for acting on the environment to the knowledge-based components. The interfaces with components outside a SADAS are isolated inside the converter and notifier components and follow an observer pattern [59]. These components also perform the translations between the SADAS-ML and the formats of the external components. Agents are introduced to manage these components and reason on their information.

The knowledge management is implemented with a rule-based social engine. Properties play the role of rules to check and derive information. The engine implements a pattern-matching algorithm that looks for instances of a query (i.e., a SADAS-ML specifications) among the available information.

The SADAS framework has been tested in several experiments. The case study (see Section 4.1) uses it to integrate two existing systems, an ITS to control the traffic lights in an area [15] and an obstacle detection system for VANs [16]. The proposed SADAS abstracts their information and uses its derivations to improve the planning of lights and the anticipation of potential movements of obstacles. The models allow most of its knowledge to be specified graphically, with only some conditions in process snippets. As stated before, modifications of this behavior mainly require changing the considered traffic social properties. Only when changes affect input data (e.g., from sensors) or acting outside the SADAS are new or modified interface components needed. Nevertheless, these interface components can be reused in projects. Similar findings appear in the other experiments reported, which consider other settings such as smart roads [40] and people’s driving behavior [37].

The approach still has several open issues, including some that already appeared in the paper. First, the SADAS-ML needs to include additional primitives. The experiments already required the introduction of new types (e.g., the information type wandering) and tagging (e.g., type of area). When these needs are recurrent, they point to potential new primitives. Another issue is the representation of decision-making. Currently, it appears as a produce-consume process on information, but there are no graphical ways to represent steps or preferences. For more complex processes, the specification must rely on snippets, which are less user-friendly for non-experts. Second, the use of the framework requires the development of guidelines for new users. Aspects such as choosing between alternative ways of modelling, or reviewing the knowledge basis, require training. Third, and following the previous points, additional tool support is required, in particular, to check the consistency of knowledge. This can be achieved with the available mechanisms through the introduction of meta-properties for validation. Finally, further experimentation is required. The framework has been tested with several existing studies, but there are still multiple traffic problems to check, such as rule breaking, incidents, autonomous vehicles, and highways. Moreover, some decisions already taken need further validation. For instance, the choice of enumeration types for some attributes and the possibilities of qualitative reasoning with them.
